# Effects of 8 days intake of hydrogen-rich water on muscular endurance performance and fatigue recovery during resistance training

**DOI:** 10.3389/fphys.2024.1458882

**Published:** 2024-10-07

**Authors:** Kaixiang Zhou, Chaoqun Yuan, Zhangyuting Shang, Wenhui Jiao, Yubo Wang

**Affiliations:** ^1^ College of Physical Education and Health Science, Chongqing Normal University, Chongqing, China; ^2^ College of Sports and Health, Chengdu University of Traditional Chinese Medicine, Chengdu, Sichuan, China; ^3^ College of Physical Education and Health Management, Chongqing University of Education, Chongqing, China; ^4^ China Institute of Sport and Health Science, Beijing Sport University, Beijing, China

**Keywords:** hydrogen-rich water, muscular endurance, countermovement jump, total quality recovery scale, visual analog scale

## Abstract

**Background:**

Exercise-induced oxidative stress and inflammation can impair muscular function in humans. The antioxidant and anti-inflammatory properties of molecular hydrogen (H_2_) highlight its potential to be as an effective nutritional supplement to support muscular function performance in healthy adults. However, the effects of H_2_ supplementation on muscular endurance performance in trained individuals have not been well characterized. This study aimed to assess the effects of intermittent hydrogen-rich water (HRW) supplementation before, during, and after resistance training on muscular endurance performance, neuromuscular status, and subjective perceptual responses after a 48-h recovery period.

**Methods:**

This randomized, double-blinded, placebo-controlled cross-over study included 18 trained men aged 19.7 ± 0.9 years. Participants in this study were instructed to consume 1,920 mL of HRW or pure water (Placebo) daily for 7 days. Additionally, participants were required to supplement with HRW or pure water five times during the training day (1,260 mL total). This included drinking 210 mL 30 min and 1 min before training, 210 mL between training sets, 210 mL immediately after training, and 420 mL 30 min into the recovery period. Participants performed half-squat exercises with the load set at 70% of one repetition maximum for six sets (half-squat exercise performed to repetitions failure each set). We measured the power output and number of repetitions in the free barbell half-squat used to assess muscular endurance performance in participants. The countermovement jump (CMJ) height, total quality recovery scale (TQRS), and muscle soreness visual analog scale (VAS) scores were measured to assess fatigue recovery status after training, as well as at 24 and 48 h of recovery.

**Results:**

The total power output (HRW: 50,866.7 ± 6,359.9W, Placebo: 46,431.0 ± 9,376.5W, p = 0.032) and the total number of repetitions (HRW:78.2 ± 9.5 repetitions, Placebo: 70.3 ± 9.5 repetitions, p = 0.019) in the H_2_ supplemented group were significantly higher than in the placebo group. However, there was no statistically significant difference (p< 0.05) between the H_2_ and placebo groups in CMJ, TQRS, and VAS.

**Conclusion:**

Eight days of intermittent HRW intake could significantly improve muscular endurance performance in trained individuals, making it a promising strategy for athletes or fitness enthusiasts looking to boost muscular endurance during resistance training or competitions. However, it should be noted that HRW intake alone may not be adequate to accelerate recovery from muscle soreness or fatigue following high-intensity training.

## 1 Introduction

Muscular endurance is the ability of a muscle to perform continuous contractions under sub-limit loads, and it is regarded as an essential component not only of athletic performance in sports but also for activities of daily living that require repetitive work (Bemben, 1998; Dere and Alemdaroğlu-Gürbüz, 2024; Varjan et al., 2024; Daniels and Howie, 2024). Evidence indicates that resistance training positively changes mitochondria and microvasculature, improving participants’ muscular endurance (Kon et al., 2010; Groennebaek et al., 2018). However, multiple sets of resistance training can cause muscle tissue damage, increased creatine kinase, and decreased neuromuscular status, leading to exercise fatigue and difficulty maintaining specific power output and completing additional repetitions (MacIntyre et al., 1995; González-Hernández et al., 2021; Pareja-Blanco et al., 2017). Additionally, intense resistance training induces delayed onset muscle soreness (DOMS), causing participants to experience discomfort in their skeletal muscles for 24–48 h after exercise (Stauber, 1989; McQuilliam et al., 2023). It has shown that the underlying mechanisms leading to muscle function impairment and exercise fatigue are localized inflammatory responses to leukocyte aggregation in injured muscle tissue, apoptosis, and reactive oxygen species (ROS) (Close et al., 2005; Carrì et al., 2015; Çakir-Atabek et al., 2019; Powers and Jackson, 2008). Therefore, efforts have been put on exploring potential anti-inflammatory and antioxidant approaches, which can thus help develop appropriate strategies to enhance muscle endurance performance .

In recent years, molecular hydrogen (H_2_) has emerged as a novel antioxidant in sports science (Kawamura et al., 2020; Ostojic, 2021; Ostojic, 2015). It offers several advantages over conventional antioxidants. H_2_ effectively reduces hydroxyl radicals (⋅OH) and peroxynitrite (ONOO-) in cells without affecting other reactive substances like superoxide (O_2_-), hydrogen peroxide (H_2_O_2_), and nitric oxide (NO) (Hong et al., 2010; Ohsawa et al., 2007). Additionally, as the smallest molecule, H_2_ can quickly enter cell membranes, diffuse into organelles such as mitochondria, and enhance mitochondrial respiration, enzyme activity, and ATP production or lactate oxidation (Ostojic, 2021; Gvozdjáková et al., 2020). H_2_ can also be exhaled, minimizing side effects (Kawamura et al., 2020; Ostojic, 2015). Given these advantages, H_2_ has been suggested to enhance exercise capacity, acting as a selective antioxidant, signaling molecule, a tonic for mitochondrial bioenergetics, and/or a buffering agent (Ostojic, 2021). Several human studies have found that pre-exercise supplementation with hydrogen-rich water (HRW) improves endurance, repeated sprint ability, and maximal isokinetic muscle strength performance in healthy adults (Kawamura et al., 2020; Ostojic, 2021; Sládečková et al., 2024; Jebabli et al., 2023; Botek et al., 2022; Timon et al., 2021; Dong et al., 2022; Aoki et al., 2012). Our previous systematic review and meta-analysis also showed that pre-exercise H_2_ supplementation effectively increased antioxidant potential and reduced subjective fatigue (e.g., rating of perceived exertion) and blood lactate concentration during aerobic and anaerobic exercise (Zhou et al., 2023; Li et al., 2024). In a study by Botek et al. (2021), it was demonstrated that intermittent HRW supplementation before, during, and after barbell half-squats (3 sets of 10 repetitions at 70%1RM) enhanced lower extremity mobility, reduced blood lactate concentrations, and alleviated DOMS in healthy adults at 24 h (Botek et al., 2021). Sládečková et al. (2024) showed that supplementation with HRW promotes muscle recovery in elite fin swimmers after two strenuous training sessions on the same day. The available evidence implicates that short-term H_2_ supplementation may be a potential strategy to improve muscular endurance performance and reduce fatigue in trained individuals. However, according to our previous systematic review (Zhou et al., 2024), no direct evidence currently supports the efficacy of short-term H₂ supplementation in enhancing muscular endurance performance or improving fatigue recovery within the first 48 h post-training in trained individuals.

Based on the literature (Sládečková et al., 2024; Botek et al., 2021; Javorac et al., 2019) and our previous studies (Dong et al., 2022; Aoki et al., 2012; Zhou et al., 2023; Li et al., 2024; Hong et al., 2022; Dong et al., 2024), we expected that HRW administration would positively affect muscle contraction function and muscle fatigue compared to placebo during muscular endurance training and up to 48 h of recovery. In this regard, we hypothesized that participants would significantly increase barbell half squat power and repetitions, increase vertical jump height and subjective fatigue recovery, and decrease Visual Analogue Scale (VAS).

## 2 Materials and methods

### 2.1 Participants

This study included 18 male students of the Faculty of Physical Culture with the following characteristics (mean ± SD): age: 19.7 ± 0.9 years (range 18–21 years); body weight: 69.7 ± 9.2 kg; body height: 176.7 ± 6.3 cm; body fat = 12.0 ± 3.8%; 1RM half squat = 136.7 ± 20.9 kg (relative strength ratio:2.4 ± 0.4). All participants were healthy, medication-free, non-smokers, did not take any dietary supplements, and performed resistance training at least three times a week. The study was approved by the Ethics Committee of Sports Science Experiment of Beijing Sport University (No. 2022214H).

### 2.2 Procedures

**FIGURE 1:**
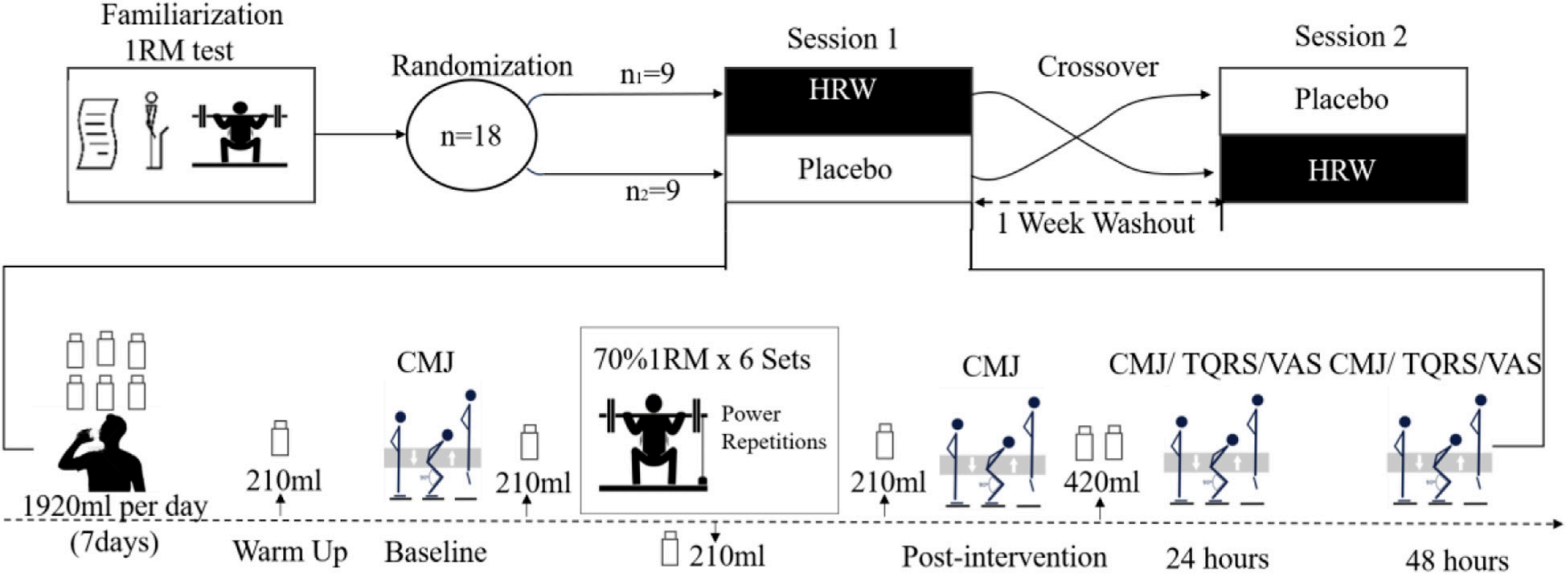
Overview of the experimental protocol sessions. 1RM, One Repetition Maximum; CMJ, Countermovement Jump; VAS, Visual Analog Scale; TQRS, Total Quality Recovery Scale.

The study was a double-blind, cross-over design with administration of HRW and placebo randomized and counterbalanced. The experimental protocol consisted of a process familiarization, a one repetition maximum (1RM) half-squat test, and two sessions with a one-week washout period (Figure 1). The participants were informed about the experimental procedure and signed an informed consent form before the 1RM half-squat test. During the experiment, participants were instructed to avoid caffeinated beverages, such as coffee or tea, and other substances that may affect physiological and perceptual outcomes. Participants were also instructed to avoid high-intensity physical activity during the experiment. To avoid environmental and diurnal variations, the intervention and tests were performed in an indoor physical training center (indoor temperature 24°C) between 13:30 and 16:30. The first session proceeded 72 h after the 1RM test. In this session, participants were randomly divided into two groups, HRW (n1 = 9) or placebo (n2 = 9), and they then followed each task scheduled in the experimental research protocol (Figure 1). Order of HRW or placebo consumption was randomized utilizing lots that included an equal number of two colored strips (yellow and green) to represent either HRW or placebo consumption first. While blinded, participants drew one strip. There was then a 1-week washout period before the second session where the beverage consumption was reversed before performing the same resistance training protocol. The coach instructed the participants to consume the same diet and not make any changes in their diet throughout the study. Participants ate together in the training center cafeteria, ensuring the food was the same.

#### 2.2.1 Anthropometric measurement

Body height and body mass were measured using a digital weighing scale DSZN-M-101A (Dashu Intelligent Technology Co., Tianjin, China). Percent body fat was determined using bioimpedance analysis (Inbody770, South Korea).

#### 2.2.2 One repetition maximum test (1RM test)

The 1RM test is a classic method for evaluating dynamic maximal muscular strength (Earle, 2008). Prior to the 1RM test, participants engaged in a 10-min warm-up on a cycle ergometer, followed by 10 min of dynamic stretching of the lower limb muscles and a set of 8–12 repetitions of a free barbell half-squat exercise at an anticipated weight equivalent to 50% of their 1RM. After a 3-min rest, participants performed four repetitions at 70%–75% of the anticipated 1RM, followed by another 3-min rest and 2-3 additional repetitions at 85%–90% of the anticipated 1RM. Following a 4-min rest period, the information gleaned from the third set was utilized to determine the final weight of each individual’s 1RM(35). Participants were required to perform each barbell half-squat in a 3/0/x/0 movement tempo following a movement rhythmizer. 3/0/x/0 denotes a 3-s eccentric phase, no intentional isometric pause during the transition phase, a concentric phase as fast as possible, and no pause between the completion of the concentric phase and the beginning (eccentric phase) of the next repetition.

#### 2.2.3 Resistance training protocol

Prior to resistance training, all participants performed a 10-min warm-up on a cycle ergometer at 60 rpm, followed by 10 min of dynamic stretching and one set of free barbell half-squats (6–8 repetitions with 50%1RM) with a 3-min rest before resistance training. Resistance training consisted of six free barbell half squats with a fixed load (70%1RM) with 5 min rest between sets. Participants were required to train to repetitions failure (inability to complete the concentric phase of a repetition) for each set. The half-squat training was performed in a half-squat rack (Technogym, Shanghai, China). They then squatted to a depth of 90° angle to the knees and returned to the starting position. Two strength coaches stood at each end of the barbell to ensure that the participants completed each half-squat correctly and safely.

#### 2.2.4 Protocol of HRW administration

The HRW was prepared using an electrolysis device (Zhiheng Hydrogen Health Technology Co., Ltd., Fuzhou, China) with a transparent Tritan™ tank body and an electrolysis generator. Participants were unable to identify HRW and placebo (purified water) due to the identical appearance of HRW and placebo and the colorless, odorless, and tasteless of H_2_. The chemical properties of HRW (pH:7.9; Oxidation reduction potential: 650mv; Temperature:22°C) and placebo (pH:7.6; Oxidation reduction potential: +167mv; Temperature:22°C) were measured using a pH/oxidation-reduction potential/temperature meter (169 E, Shenzhen Yiyi Yiqi Science and Technology Co., Ltd., China). The concentration of H_2_ in the HRW was 1,600 ppb, according to the manufacturer’s instructions. Based on previous studies (Timon et al., 2021; Botek et al., 2021), we developed the HRW supplementation protocol for this study, as there is no gold standard for using H_2_ to enhance muscular endurance performance. Previous studies (Timon et al., 2021; Botek et al., 2021) have shown that this H_2_ supplementation protocol meets individual H_2_ intake requirements without causing adverse effects. Participants were instructed to drink 1,920 mL (six bottles of HRW or PW according to a crossover double-blind design) of water per day (7 days in total), and all of the water had to be drunk immediately after the hydrogenation process in order to maintain hydrogen concentration levels and avoid deterioration of oxidation reduction potential. Additionally, participants were required to supplement with five doses of HRW or PW during each training session, specifically 210 mL at 30 min and 1 min before training, 210 mL during the middle of training, another 210 mL immediately after training, and 420 mL of HRW at 30 min during the recovery period.

#### 2.2.5 Muscular endurance performance

Muscular endurance performance was defined as the power output and maximum number of repetitions performed by participants during resistance training with a load of 70% of their current one-repetition maximum (1RM) (Fliss et al., 2022; Vieira et al., 2021). The primary outcomes of muscular endurance performance were single-set power output and six-set total power output completed by participants in the free barbell half squat, and the secondary outcomes were the single-set number of repetitions and six-set total number of repetitions completed. A linear position transducer measured the power output and number of repetitions (Linear encoder kit, Chronojump, Spanish). The linear position transducer consists of a floor unit consisting of a spring-powered retractable wound on a cylindrical spool coupled to the shaft of an optical encoder (Drinkwater et al., 2007). The floor unit is placed on the floor perpendicular to the right collar of the barbell. The other end of the cable was attached vertically to the barbell (immediately proximal to the right collar) with a Velcro strap for data acquisition (Grgic et al., 2020). Previous studies have shown the linear position transducer to be reliable for measuring power during resistance training (Grgic et al., 2020; Orange et al., 2020; Askow et al., 2018; García-Ramos et al., 2018).

#### 2.2.6 Fatigue

Fatigue could be generally defined as a decrease in physical performance related to a rise within the real/perceived difficulty of a task or exercise, as well as the inability of the muscles to keep up with the specified level of strength during exercises (Gandevia, 2001; Abd-Elfattah et al., 2015). This study used repetitions to failure exercise to ensure that participants’ skeletal muscles were fatigued (González-Hernández et al., 2021). The primary outcomes of fatigue were the height of the Countermovement Jump test (CMJ) at baseline (CMJ_0_), 5 min (CMJ_5_), 24 h (CMJ_24_), and 48 h (CMJ_48_) post-training. The secondary outcomes were the Total Quality Recovery Scale (TQRS) and Visual Analogue Scale (VAS) at 24 h (VAS_24_/TQRS_24_) and 48 h (VAS_48_/TQRS_48_) post-training.

##### 2.2.6.1 Countermovement jump test (CMJ)

The CMJ test is an appropriate method for monitoring neuromuscular fatigue in participants (Gathercole et al., 2015; Claudino et al., 2017). Before the test, the participants completed a warm-up according to the resistance training protocol. After a 3-min rest, the participants performed three one-repetition maximal effort CMJs, with a 30-s rest between each jump. The starting position for the CMJ required the body to stand upright on a jump mat (Contact Platform Kit, Chronojump, Spanish), with the hands on the hips to avoid swinging the arms. Then, a fast downward movement was performed to an optimal position (approximately 90° at the knee), followed by a fast vertical movement upward as forceful as possible. The maximum height of the CMJs was used for data analysis. Participants were not required to perform an additional warm-up during the CMJ test 5 minutes after resistance training.

##### 2.2.6.2 Total quality recovery scale (TQRS)

The TQRS is used to assess perceived and action recovery (Kenttä and Hassmén, 1998). The participants rated their recovery over the previous 24 and 48 h using the question, “What is your condition now?” The TQRS ranged from 0 (very, very poor recovery) to 10 (very, very good recovery) (Kenttä and Hassmén, 1998).

##### 2.2.6.3 Visual Analogue Scale (VAS)

DOMS occurs 24–48 h following a resistance training session. Evidence shows that training with sore muscles while trying to sustain a high load can lead to overreaching (Meeusen et al., 2013). The VAS is commonly used to measure DOMS (Lau et al., 2015; Nosaka et al., 2002).The VAS was used to measure lower limb muscle pain at 24 and 48 h after resistance training. The VAS is a 100 mm long horizontal line, with 0 indicating “no pain” and 100 indicating “worst pain imaginable” (Lau et al., 2015).

### 2.3 Statistical analysis

Data were presented as arithmetic mean ± SD. The normality of data was tested using the Kolmogorov-Smirnov test. This experiment utilized a repeated-measures design that allowed for repeated measurements at different time points within the same group of subjects, necessitating repeated-measures ANOVA to handle this correlational data. We used a two-way (time*group) repeated-measures ANOVA to evaluate changes in muscular endurance performance (power output, repetitions) during six-set training and fatigue recovery (CMJ, TQRS, and VAS) from immediate to 48 h post-training. Post hoc analyses were performed using the Bonferroni test. Repeated-measurement data was needed to satisfy Mauchly’s test (p > 0.05), otherwise an epsilon (ε) correction was required. If no interaction between time and group was observed, the data were analyzed for the main effect, and then effect sizes [partial eta-squared ( $\eta_{p}^{2}$ )] were calculated, where effect sizes were categorized as trivial ( $\eta_{p}^{2}$ <0.01), small (0.01≤ $\eta_{p}^{2}$ <0.06), moderate (0.06 $\leq \eta_{p}^{2}$ <0.14), and large ( $\eta_{p}^{2}$ ≥0.14) effects (Botek et al., 2021). In contrast, if an interaction between time and group was detected, the data were analyzed for simple effects and effect sizes were calculated (Cohen’s d). The effect size could be useful for calculating performance changes following a training program (Hopkins, 2004). Cohen’s d was calculated as (experimental group mean - control group mean)/control group standard deviation. Cohen’s d was classified as trivial effect (d < 0.2), small effect (0.2 ≤ d < 0.5), moderate effect (0.5 ≤ d < 0.8), and large effect (d ≥ 0.8) (Cohen, 1988). Additionally, we analyzed the overall effect of HRW on participants’ total power output and total repetitions using a paired-sample t-test and Cohen’s d, considering the training session as a whole. Differences in means were also expressed using 95% confidence intervals (CIs). For all tests, p < 0.05 was considered statistically significant. All analyses were performed using the SPSS statistical package (version 25.0, IBM Statistics, Chicago, IL).

## 3 Results

### 3.1 Effect of H_2_ on muscular endurance

#### 3.1.1 Power output

There was no interaction between time and group (F = 1.233, p = 0.306). Time main effects showed a significant decrease (F = 48.222, p < 0.001, $\eta_{p}^{2}$ = 0.739, large effect) in power output in both the HRW and placebo groups from the 1st to the 6th training set. Meanwhile, group main effect showed that the H_2_ supplementation group performed significantly higher power output than the placebo group (F = 5.435, p = 0.032, $\eta_{p}^{2}$ = 0.242, large effect) (Table 1).

**TABLE 1:** Effect of H_2_ supplementation on fatigue.

Variable	Group	Baseline	P5 min	P24 h	P48 h	Time factor		Water factor		Interaction
						p	$\eta_{p}^{2}$	p	$\eta_{p}^{2}$	p
CMJ (cm)	HRW	51.0 ± 7.2	48.2 ± 7.5	49.7 ± 6.9	51.4 ± 6.6	<0.001	0.265	0.621	0.007	0.212
	Placebo	50.7 ± 6.6	47.5 ± 7.0	48.6 ± 6.4	49.0 ± 7.3					
VAS (mm)	HRW	—	—	37.2 ± 21.6	26.5 ± 21.0	<0.001	0.394	0.380	0.023	0.975
	Placebo			41.9 ± 15.1	30.9 ± 19.0					
TQRS	HRW	—	—	6.8 ± 1.8	7.9 ± 1.9	<0.001	0.671	0.099	0.078	0.296
	Placebo			5.1 ± 1.8	7.1 ± 1.2					

p, statistical significance; $\eta_{p}^{2}$ , partial eta-squared effect size; CMJ, countermovement jump test; VAS, visual analog scale; TQRS, Total Quality Recovery Scale.

— = No measurements were taken.

**FIGURE 2:**
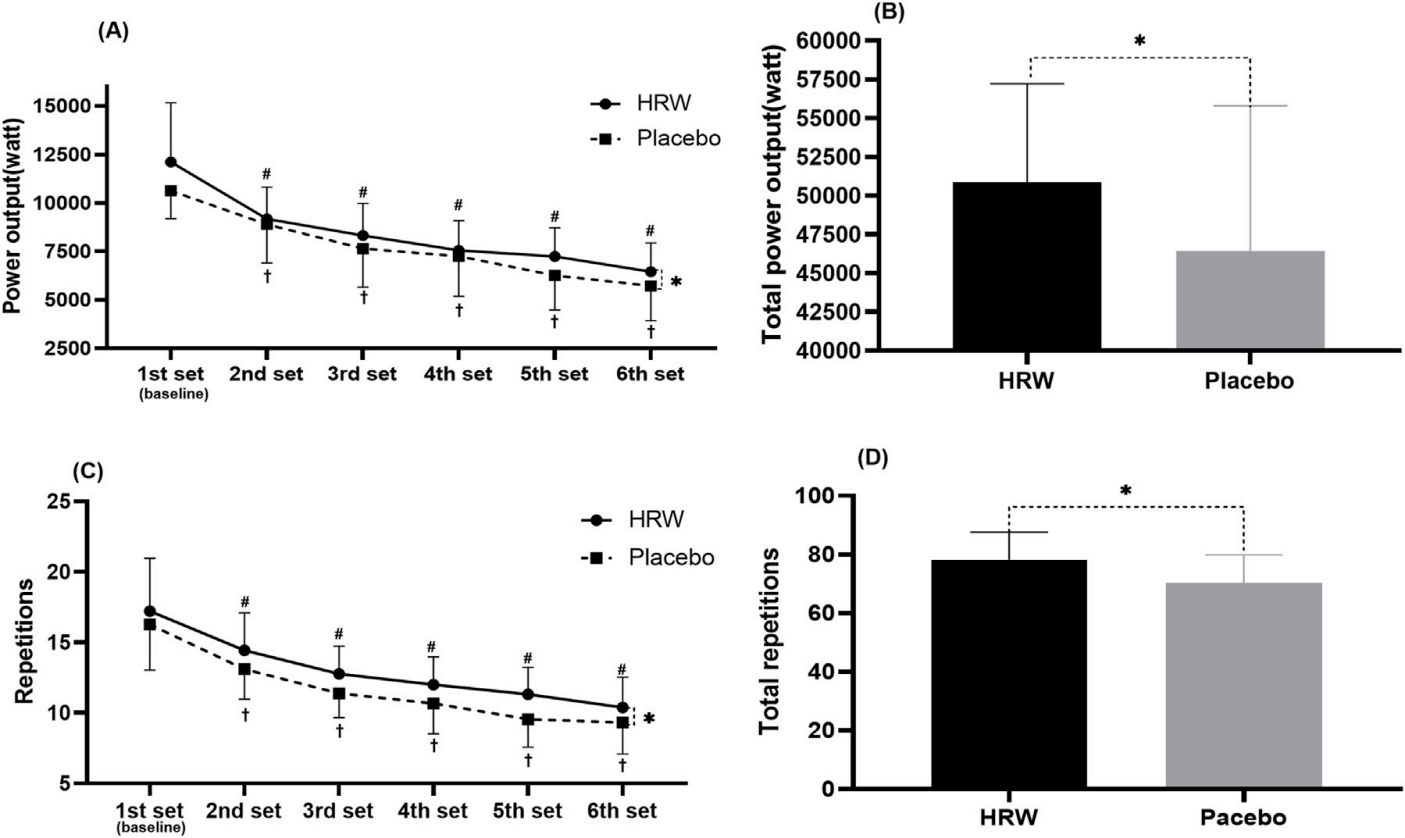
Effect of hydrogen rich water (HRW) on muscular endurance performance. Values are presented as the mean and standard deviation. **(A)** Effect of HRW on single-set power output. **(B)** Effect of HRW on six-set total power output. **(C)** Effect of HRW on single-set number of repetitions. **(D)** Effect of HRW on six-set total number of repetitions. * = statistically significant (p < 0.05) difference between hydrogen rich water and placebo at the same time. ^#^ = statistically significant (p < 0.05) for the hydrogen rich water group compared to baseline. ^†^ = statistically significant (p < 0.05) for the placebo group compared to baseline.

H_2_ supplementation significantly enhanced total power output compared to placebo in six sets of free barbell half-squat exercises (HRW: 50,866.7 ± 6,359.9W, Placebo: 46,431.0 ± 9,376.5W, 95%CI:421.4∼8,449.9, p = 0.032, d = 0.47, small effect) (Figure 2).

#### 3.1.2 Repetitions

There was no interaction between time and group (F = 0.244, p = 0.819). Time main effects showed a significant decrease (F = 51.357, p < 0.001, $\eta_{p}^{2}$ = 0.751, large effect) in repetitions in both the HRW and placebo groups from the 1st to the 6th training set. Meanwhile, group main effect showed that the H_2_ supplementation group performed significantly more repetitions than the placebo group (F = 6.709, p = 0.019, $\eta_{p}^{2}$ = 0.283, large effect).

H_2_ supplementation significantly increased total repetitions compared to placebo in six sets of free barbell half-squat exercises (HRW:78.2 ± 9.5 repetitions, Placebo: 70.3 ± 9.5 repetitions, 95%CI:1.5∼14.2, p = 0.019, d = 0.82, large effect) (Figure 2).

### 3.2 Effect of H_2_ on fatigue

#### 3.2.1 Countermovement jump

**FIGURE 3:**
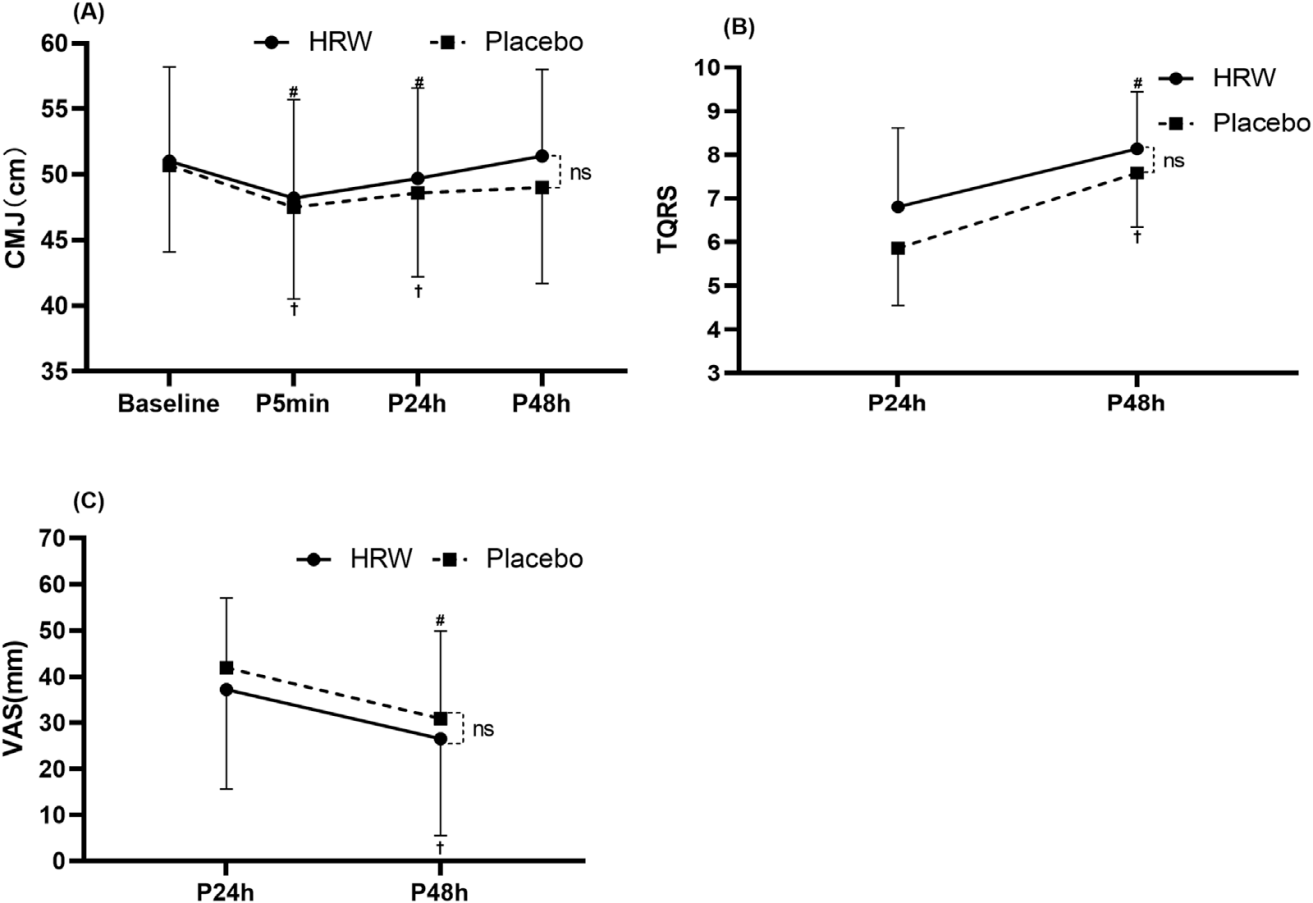
Effect of hydrogen rich water (HRW) on fatigue. **(A)** Effect of HRW on Countermovement Jump height (CMJ). **(B)** Effect of HRW on Total Quality Recovery Scale (TQRS). **(C)** Effect of HRW on Visual Analog Scale (VAS). Values are presented as the mean and standard deviation. * = statistically significant (p < 0.05) difference between hydrogen rich water and placebo at the same time. ns = no statistically significant difference was found between hydrogen-rich water and placebo at the same time (p > 0.05). ^#^ = statistically significant (p < 0.05) for the hydrogen rich water group compared to baseline. ^†^ = statistically significant (p < 0.05) for the placebo group compared to baseline.

There was no interaction between time and group (F = 1.529, p = 0.212). Time main effects showed that participants in the HRW and placebo groups had statistically significant (F = 12.241, p < 0.001, $\eta_{p}^{2}$ = 0.265, large effect) CMJ heights after resistance training compared to baseline (CMJ_0_). Specifically, there was a significant decrease in CMJ_5_ (p < 0.001, 95% CI: 1.28–44.6) and CMJ_24_ (p = 0.014, 95% CI: 0.25–3.11) compared to baseline (CMJ_0_), but no statistically significant difference between CMJ_48_ and baseline (CMJ_0_) (p > 0.05, 95% CI: 0.98–2.32) in the HRW and placebo groups. Group main effect showed that H_2_ supplementation failed to significantly increase CMJ height at 5 min, 24 and 48 h compared to placebo (F = 0.249, p = 0.621, $\eta_{p}^{2}$ = 0.007, trivial effect) (Figure 3).

#### 3.2.2 Total quality recovery scale

There was no interaction between time and group (F = 1.126, p = 0.296). Time main effect showed a significant increase (F = 69.493, p < 0.001, $\eta_{p}^{2}$ = 0.671, large effect) in TQRS scores at 24 and 48 h after training in both the HRW and placebo groups. However, group main effect showed that H_2_ supplementation failed to significantly increase TQRS scores at 24 and 48 h compared to placebo (F = 2.882, p = 0.099, $\eta_{p}^{2}$ = 0.078, moderate effect) (Figure 3).

#### 3.2.3 Visual analog scale

There was no interaction between time and group (F = 0.001, p = 0.975). Time main effect showed a significant decrease (F = 22.096, p < 0.001, $\eta_{p}^{2}$ = 0.394, large effect) in VAS scores at 24 and 48 h after training in both the HRW and placebo groups. However, group main effect showed that H_2_ supplementation failed to significantly reduce VAS scores at 24 and 48 h compared to placebo (F = 0.792, p = 0.380, $\eta_{p}^{2}$ = 0.023, small effect) (Figure 3).

## 4 Discussion

This study aimed to assess the effects of HRW supplementation in trained individuals for 8 days (including the training day) on muscular endurance performance during training, neuromuscular status, and perceptual responses during the recovery period (24 h, 48 h). This study revealed that 8 days of HRW supplementation effectively enhanced muscular endurance performance in trained individuals but failed to promote fatigue recovery significantly after training.

In our resistance training protocol, all half-squat sets were repetitions to failure by the participant at maximal voluntary effort (single set completion time over 30 s), with a five-minute passive recovery time between sets. In the case of high-intensity exercise, when the phosphocreatine (PC) system is depleted within the first few seconds, anaerobic glycolysis becomes the primary metabolic pathway, usually leading to blood lactate accumulation and exercise fatigue (Brooks, 2018). Studies have shown that HRW supplementation reduces post-exercise blood lactate concentrations and subjective fatigue (Zhou et al., 2023). This study supports Botek et al. (2021) recommendation for practical application that acute ingestion of HRW is promising as an effective hydration strategy for athletes to improve lower limb muscular endurance performance. Trainers could utilize HRW supplementation as a muscular endurance performance enhancer for athletes during the phase of muscular endurance training, typically lasting 7 days. This improvement may be attributed to the stimulating effect of hydrogen molecules on mitochondrial oxidative phosphorylation, which in turn enhances the antioxidant potential of the human body during high-intensity exercise (Li et al., 2024; Murakami et al., 2017; Cheng et al., 2023). Additionally, counteracting exercise-induced acidosis is a well-known routine for improving exercise performance and avoiding fatigue (Lancha Junior et al., 2015). Studies have shown that HRW can increase fasting and post-exercise blood pH during exercise or affect acid-base balance and metabolic fatigue (Botek et al., 2021; Ostojic, 2012; Ostojic and Stojanovic, 2014; Alharbi et al., 2022). H_2_ also could reduce intracellular reactive oxygen species (ROS) levels and thus enhance muscle contractile function (Powers and Jackson, 2008; Shibayama et al., 2020). For instance, a study on soccer players showed that administering three successive doses of 500 mL HRW before high-intensity aerobic exercise increased the mean power frequency of skeletal muscles during subsequent strength tests (Aoki et al., 2012). One study found that inhaling H_2_ gas before exercise can reduce fatigue during high-intensity exercise by maintaining high prefrontal cortex activation (Hong et al., 2022). The dose-response relationship between H_2_ and physical performance has yet to be established, making it difficult to determine the most appropriate dosage supplementation protocol for H_2_ in improving muscular endurance performance (Zhou et al., 2024). This study used a strategy of intermittent multi-dose HRW supplementation for eight consecutive days. Studies have shown that HRW supplementation for consecutive days effectively improves aerobic and anaerobic capacity in trained athletes (Timon et al., 2021; Da et al., 2018). One potential reason for this is that prolonged (7 days or more) intake of H_2_ during resistance training may contribute to increased mitochondrial biogenesis and endogenous antioxidant systems, as well as improved muscle endurance performance (Li et al., 2024; Botek et al., 2021). However, one study showed that HRW supplementation for two consecutive weeks did not significantly enhance the biological antioxidant potential of college students (Hori et al., 2020). This heterogeneity may stem from differences in participants’ training status. Timon et al. (2021) showed that the participants’ training status influenced the benefits of HRW and that 7 days of HRW intake increased anaerobic capacity in trained cyclists but had no effect on untrained subjects. Future studies should focus on directly measuring the antioxidant potential of HRW supplementation over consecutive days in trained individuals, especially athletes. For instance, researchers could use the oxidative stress parameters recommended in the Kayacan et al. study to directly assess the antioxidant capabilities during exercise (Kayacan et al., 2022; Kayacan et al., 2018; Kayacan et al., 2019). Mikami et al. (2019) reported that most H_2_ could be maintained in the body for 30–40 min after HRW administration. Therefore, this study utilized HRW supplementation before, during, and after exercise to ensure H_2_ concentrations in the participants somewhat. More research is still necessary to explore the dosage and timing of HRW supplementation to enhance exercise performance further.

Contrary to expectations, this study did not find HRW effective in promoting neuromuscular status (CMJ) and subjective fatigue recovery in participants after high-intensity resistance training. These results are consistent with those of Botek et al. (2021), who found no significant difference between HRW and placebo in Creatine kinase concentrations, VAS, and CMJ, during 24-h post-exercise recovery. One potential reason for this finding is that the H_2_ content of a one-time supplement of HRW (e.g., 420 mL) is insufficient to restore the oxidative stress status of skeletal muscle after high-intensity resistance training without significantly restoring neuromuscular status (Li et al., 2024). The CMJ performance duration is 1–2 s, unlike the longer duration half squat (>30 s), and is mainly dependent on the energy of the ATP-PC system and the elastic potential of the muscle (stretch-shortening cycle). Studies have shown that drinking 1,260 mL of HRW before and during exercise may enhance the endogenous antioxidant capacity to respond to the intensity dependent, mitochondrial production of ROS, reduce oxidative stress, and enhance mitochondrial ATP production (Çakir-Atabek et al., 2019; Murakami et al., 2017; Radak et al., 2017). However, drinking HRW after intense exercise might not be compelling enough to improve neuromuscular status. For example, one study showed that short-term HRW supplementation did not significantly improve the surface electromyographic parameters (e.g., RMS amplitude, high-frequency power, low-frequency power) of the quadriceps muscle after resistance training in participants (Botek et al., 2021). Therefore, in practice, participants may need to continuously supplement HRW during fatigue recovery (e.g., 24 h, 48 h after training) to achieve a significant effect. Additionally, the training status of the participants in this study may also be an essential factor contributing to the failure of HRW supplementation after resistance training to relieve skeletal muscle pain and subjective fatigue recovery. The results indicated that the HRW and placebo groups had returned to their baseline levels of countermovement jump (CMJ) height 48 h after training. This is because DOMS in trained participants may not be severe after resistance training. This means that the neuromuscular status of the participants could be wholly recovered 48 h after the training. Study has shown that muscle fatigue in trained individuals cloud be recovered within 48 h after resistance training (Morán-Navarro et al., 2017). Kawamura et al. (2020) suggested that short-term intake of HRW, an alternative recovery procedure, is unlikely to reduce inflammation and oxidative stress effectively after high-intensity exercise. As a result, acute intake of HRW alone after high-intensity resistance training may not be an efficient recovery strategy. Trainers may include short-term HRW as part of a varied nutritional approach or as an alternative to hydration. Considering the limitations of hydrogen concentration in HRW, fatigue recovery may be more favorable if participants directly inhale a more concentrated hydrogen-rich gas (HRG). One study has shown that inhalation of HRG during the post-exercise recovery period reduces systemic oxidative damage, thus facilitating improved lower limb performance (Shibayama et al., 2020). Future studies should directly compare HRW and HRG to promote fatigue recovery in participants.

There are some limitations in the application of HRW. This study did not establish an optimal H_2_ supplementation protocol based on individual H_2_ dynamics. Few papers have reported changes in H_2_ concentration in the living body after HRW intake (Kawamura et al., 2020). Therefore, administration immediately before and during exercise may be necessary to obtain an acute H_2_ effect. In addition, participants were only supplemented with H_2_ for 8 days, which may not clarify the long-term effects of H_2_ on muscular endurance performance or safety. Studies have shown that excessive intake of conventional antioxidants such as vitamins C and E can inhibit redox-sensitive signaling pathways and interfere with physiological adaptations to exercise training (Gomez-Cabrera et al., 2015; Kawamura and Muraoka, 2018). Future studies should promptly clarify the effects of long-term H_2_ supplementation on physiological adaptations induced by prolonged exercise training and its safety. This study utilized a randomized crossover trial, which could not wholly avoid the influence of the training trace effect on the muscle soreness score. Future studies should use randomized controlled trials to explore the long-term effects of HRW supplementation on muscle function and structure.

## 5 Conclusion

Intermittent intake of HRW for 8 days could significantly enhance muscular endurance performance in trained individuals, making it a promising strategy for athletes or fitness enthusiasts seeking to improve muscular endurance during resistance training or competitions. Based on our findings, HRW administration is usually recommended before and during muscular endurance training. However, it should be noted that HRW intake alone may not be sufficient to promote recovery from muscle soreness or fatigue after muscular endurance training. Future RCTs with rigorous designs are needed to help obtain more definitive conclusions on the long-term effects of HRW on muscular function performance in trained individuals.

## Data Availability

The raw data supporting the conclusions of this article will be made available by the authors, without undue reservation.
